# Supplementary material for: Hot spot prediction in protein-protein interactions by an ensemble system
Source: BMC Syst Biol. 2018 Dec 31;12(Suppl 9):132. doi: 10.1186/s12918-018-0665-8 (PMC6311905; doi:10.1186/s12918-018-0665-8)
Supplement: Supplementary file 2 — All relevant MATLAB code. A simple Matlab implement of our predictor. (ZIP 19,742 kb) [file 12918_2018_665_MOESM2_ESM.zip › Additional file 2/Read ASA/ASA website/SKEMPI/3BK3C.htm]

 NetSurfP - Protein Surface Accessibility and Secondary Structure Predictions

  

|  |  |  |
| --- | --- | --- |
|  |  | NetSurfP - Protein Surface Accessibility and Secondary Structure Predictions  Technical University of Denmark |

  


---

```
# For publication of results, please cite:
# A generic method for assignment of reliability scores applied to solvent accessibility predictions.
# Bent Petersen, Thomas Nordahl Petersen, Pernille Andersen, Morten Nielsen and Claus Lundegaard
# BMC Structural Biology 2009, 9:51 doi:10.1186/1472-6807-9-51
#
# Column 1: Class assignment - B for buried or E for Exposed - Threshold: 25% exposure, but not based on RSA
# Column 2: Amino acid
# Column 3: Sequence name
# Column 4: Amino acid number
# Column 5: Relative Surface Accessibility - RSA
# Column 6: Absolute Surface Accessibility
# Column 7: Z-fit score for RSA prediction
# Column 8: Probability for Alpha-Helix
# Column 9: Probability for Beta-strand
# Column 10: Probability for Coil
E W  3BK3C                  1    0.713 171.501  -0.871   0.003   0.003   0.994
E L  3BK3C                  2    0.430  78.770  -0.459   0.020   0.205   0.775
B I  3BK3C                  3    0.225  41.607  -1.169   0.022   0.359   0.619
E T  3BK3C                  4    0.494  68.546  -0.409   0.021   0.279   0.699
E G  3BK3C                  5    0.528  41.554  -1.218   0.019   0.141   0.840
E T  3BK3C                  6    0.465  64.468   1.002   0.056   0.142   0.802
E E  3BK3C                  7    0.549  95.858   0.699   0.135   0.317   0.548
E A  3BK3C                  8    0.479  52.819   0.270   0.135   0.317   0.548
E S  3BK3C                  9    0.492  57.709   1.133   0.125   0.227   0.648
B C  3BK3C                 10    0.104  14.545  -0.436   0.125   0.227   0.648
E E  3BK3C                 11    0.595 103.964   0.064   0.056   0.142   0.802
E N  3BK3C                 12    0.550  80.549  -0.526   0.052   0.084   0.864
E E  3BK3C                 13    0.602 105.152   0.435   0.018   0.088   0.893
E G  3BK3C                 14    0.424  33.353  -1.156   0.019   0.141   0.840
E E  3BK3C                 15    0.416  72.693   0.347   0.022   0.552   0.426
E V  3BK3C                 16    0.351  53.964   0.856   0.018   0.846   0.136
B L  3BK3C                 17    0.168  30.724   0.832   0.011   0.918   0.071
E H  3BK3C                 18    0.523  95.170   0.068   0.021   0.756   0.223
B I  3BK3C                 19    0.136  25.252   0.887   0.004   0.420   0.576
B P  3BK3C                 20    0.301  42.684  -0.125   0.004   0.197   0.799
E N  3BK3C                 21    0.499  73.039  -0.681   0.022   0.359   0.619
B I  3BK3C                 22    0.152  28.120   0.312   0.022   0.359   0.619
E T  3BK3C                 23    0.448  62.151  -0.487   0.021   0.279   0.699
E D  3BK3C                 24    0.548  78.909  -1.051   0.018   0.088   0.893
E N  3BK3C                 25    0.539  78.939  -0.962   0.018   0.019   0.964
B P  3BK3C                 26    0.188  26.748   0.277   0.018   0.047   0.935
B C  3BK3C                 27    0.086  12.032  -0.225   0.022   0.552   0.426
B I  3BK3C                 28    0.069  12.673  -0.103   0.021   0.756   0.223
B S  3BK3C                 29    0.165  19.350  -0.719   0.018   0.846   0.136
B C  3BK3C                 30    0.032   4.507  -0.196   0.011   0.918   0.071
B V  3BK3C                 31    0.200  30.663   0.390   0.011   0.918   0.071
B C  3BK3C                 32    0.052   7.315  -0.879   0.002   0.816   0.182
E L  3BK3C                 33    0.411  75.199   0.108   0.004   0.420   0.576
E N  3BK3C                 34    0.642  94.018  -0.526   0.018   0.088   0.893
E Q  3BK3C                 35    0.553  98.837   0.687   0.020   0.205   0.775
E K  3BK3C                 36    0.618 127.143   1.035   0.021   0.756   0.223
B A  3BK3C                 37    0.191  21.092   0.525   0.018   0.846   0.136
E E  3BK3C                 38    0.600 104.785   1.297   0.018   0.846   0.136
B C  3BK3C                 39    0.117  16.371  -0.726   0.023   0.655   0.322
E K  3BK3C                 40    0.541 111.345   0.565   0.074   0.484   0.442
E Q  3BK3C                 41    0.541  96.623   0.419   0.135   0.317   0.548
E E  3BK3C                 42    0.554  96.819   1.000   0.135   0.317   0.548
E K  3BK3C                 43    0.558 114.842   1.174   0.118   0.150   0.732
B C  3BK3C                 44    0.081  11.330  -0.787   0.113   0.043   0.844
E A  3BK3C                 45    0.515  56.808   0.253   0.113   0.043   0.844
E P  3BK3C                 46    0.582  82.657  -0.402   0.053   0.043   0.903
E L  3BK3C                 47    0.326  59.746   0.030   0.058   0.017   0.925
E A  3BK3C                 48    0.461  50.791   0.033   0.058   0.017   0.925
E E  3BK3C                 49    0.715 124.963   0.426   0.181   0.016   0.803
E D  3BK3C                 50    0.423  60.882  -0.030   0.257   0.016   0.727
B C  3BK3C                 51    0.048   6.781   0.291   0.386   0.097   0.517
E A  3BK3C                 52    0.315  34.713   0.293   0.453   0.248   0.299
B L  3BK3C                 53    0.188  34.478   0.675   0.487   0.370   0.144
B V  3BK3C                 54    0.102  15.662   0.737   0.594   0.285   0.121
B V  3BK3C                 55    0.082  12.619   0.377   0.594   0.285   0.121
E K  3BK3C                 56    0.413  84.872   0.688   0.578   0.229   0.194
E Q  3BK3C                 57    0.515  91.997  -0.193   0.428   0.171   0.402
E T  3BK3C                 58    0.451  62.540   0.061   0.191   0.086   0.723
E G  3BK3C                 59    0.583  45.906  -1.991   0.053   0.043   0.903
E A  3BK3C                 60    0.327  36.079  -0.109   0.053   0.043   0.903
B C  3BK3C                 61    0.145  20.386  -0.591   0.113   0.043   0.844
B C  3BK3C                 62    0.131  18.378   0.111   0.113   0.043   0.844
E E  3BK3C                 63    0.691 120.665   0.654   0.184   0.043   0.773
E K  3BK3C                 64    0.627 128.933   1.175   0.181   0.016   0.803
B C  3BK3C                 65    0.206  28.894  -1.780   0.115   0.016   0.868
E K  3BK3C                 66    0.703 144.628   0.673   0.053   0.043   0.903
E G  3BK3C                 67    0.937  73.710  -0.716   0.000   0.001   0.999

Explain the output.  Go back.
```

---
